# Regnase-1 and Roquin regulate inflammatory mRNAs

**DOI:** 10.18632/oncotarget.4891

**Published:** 2015-07-18

**Authors:** Takashi Mino, Osamu Takeuchi

**Affiliations:** Laboratory of Infection and Prevention, Institute for Virus Research, Kyoto University, AMED-CREST, AMED, Kyoto, Japan

**Keywords:** Chromosome Section, mRNA decay, Translation, UPF1, Cytokine

Inflammation is mediated by proinflammatory cytokines such as tumor necrosis factor (TNF) and interleukin 6 (IL-6). Innate immune cells sense pathogen infection via Toll-like receptors (TLRs) and rapidly induced production of cytokines. The expression of cytokine genes is tightly regulated at both transcriptional and post-transcriptional levels. Whereas transcription is the first step in the regulation of gene expression, post-transcriptional regulation that modifies mRNA stability and translation provides rapid and flexible control of cytokine gene and protein expression. In particular, post-transcriptional regulation is involved in resolution of inflammation and prevention of unintended tissue damage caused by excess inflammation [1]. Post-transcriptional events occur in various intracellular organelles, because eukaryotic mRNAs are in dynamic equilibrium between different subcellular locations; actively translated mRNAs can be found in polysomes, mRNAs stalled in translation initiation can accumulate in stress granules (SGs), and mRNAs targeted for degradation or translation repression can accumulate in processing bodies (PBs).

The degradation of mRNA seems to be the most efficient determinant of deactivation of inflammation, since it restricts the production of proinflammatory cytokines. Most post-transcriptional events leading to mRNA decay involve the interaction of RNA with RNA-binding proteins (RBPs). Regulatory RBPs bind to conserved *cis*-elements, including AU-rich elements (AREs) and stem-loop (SL) structures in their 3′ untranslated regions (UTRs) [1], and induce endonucleolytic cleavage or deadenylation-and decapping-mediated exonucleolytic decay of mRNAs. Recently, it has been shown that SL structures present in a set of mRNAs including *ICOS*, *OX40* and *TNF* are destabilized by Roquin [2]. Roquin harbors a ROQ domain and a CCCH-type zinc finger domain; a loss of function mutation in the Roquin ROQ domain (M199R) in mice (San) leads to the development of autoimmune disease characterized by an increase in follicular helper T cells due to high ICOS expression [3]. Roquin-mediated mRNA decay takes place in PBs or SGs by recruiting a CCR4-CAF1-NOT deadenylase complex that initiates mRNA degradation.

Regnase-1 (also known as Zc3h12a, Mcpip1) is an endonuclease critical for preventing a severe autoimmune inflammatory disease in mice by destabilizing a set of target mRNAs *via* binding to a SL structure [4-6]. Regnase-1 harbors a PIN-like RNase domain and a CCCH-type zinc-finger domain and controls a set of genes including *Il6*, *Il12p40* and *Regnase-1* itself in macrophages [4]. Regnase-1 is also essential for suppressing aberrant activation of T cells in a cell-intrinsic manner, and targets a set of genes, (e.g. *Icos*, *c-Rel*, *Ox40* and *Il2*) for degradation [6]. However, the target specificity of Regnase-1 and the molecular mechanisms by which Regnase-1 degrades its target mRNAs are not yet understood.

Our study published in *Cell*, demonstrates that although Regnase-1 and Roquin regulate an overlapping set of mRNAs *via* a common SL structure, they function in distinct subcellular locations: ribosome/endoplasmic reticulum (ER) and PBs/SGs, respectively [7]. We identified Regnase-1 and Roquin target mRNAs by RNA-immunoprecipitation sequencing (RIP-Seq) and gene set enrichment analysis (GSEA) showed that Regnase-1 target mRNAs were significantly biased toward high enrichment scores in the Roquin RIP-Seq data, indicating that the Regnase-1 and Roquin target mRNAs overlap significantly. Next, we investigated target structures of Regnase-1 binding mRNAs globally using high-throughput sequencing of RNA isolated by crosslinking immunoprecipitation (HITS-CLIP) and found that SL sequences with varying stem lengths (3-7 nucleotides) with pyrimidine-purine-pyrimidine nucleotide tri-loops preferentially associate with Regnase-1. Interestingly, this rule is consistent with that in the reported Roquin target mRNAs [2]. Indeed, Regnase-1 and Roquin destabilized the same sets of mRNAs with a target SL sequence. Taken together, these results demonstrate that Regnase-1 and Roquin recognize overlapping target mRNAs *via* the same SL structures present in their 3′ UTRs.

Despite the presence of overlapping target mRNAs, Regnase-1 and Roquin are found to degrade inflammatory mRNAs *via* spatiotemporally distinct mechanisms. Whereas Roquin localized to SGs and PBs, Regnase-1 localized to cytoplasm, ER and polysome, but not to PBs and SGs. Regnase-1 destabilized translationally active mRNAs and translation termination was required for Regnase-1-mediated mRNA decay. Furthermore, we found that Regnase-1 associated with UPF1, an RNA helicase essential for nonsense-mediated RNA decay (NMD), and UPF1 helicase activity was critical for Regnase-1-mediated mRNA decay. Whereas Regnase-1 and Roquin in part redundantly regulate their target mRNAs, Regnase-1 and Roquin tend to control the early and late phase of inflammation, respectively.

**Figure 1:**
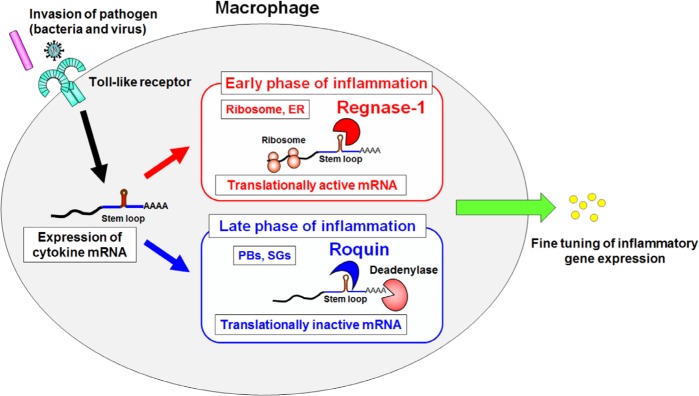
A proposed model of mRNA degradation by Regnase-1 and Roquin

Collectively, our recent study has clearly demonstrated that the post-transcriptional regulation of inflammation is controlled by Regnase-1 and Roquin in a spatiotemporally distinct manner (Figure 1). It is interesting that the machinery used for the NMD quality control system are also shared with those for the degradation of cytokine mRNAs. Although UPF1 is essential for Regnase-1-mediated mRNA decay, the molecular mechanisms how UPF1 is involved in it are still unclear. Because excess and prolonged production of cytokines leads to the onset of inflammatory diseases, prolonged stability of inflammatory cytokine mRNAs may be considered aberrant, and thus targeted by a similar mechanism of the quality control system. Strict control over the life of mRNA by RBPs is a key strategic step by which immune cells determine their phenotypes and functions, and differential regulation of Regnase-1-and Roquin-mediated mRNA degradation are thus necessary for the elaborate control of inflammation.
